# Action Costs Rapidly and Automatically Interfere with Reward-Based Decision-Making in a Reaching Task

**DOI:** 10.1523/ENEURO.0247-21.2021

**Published:** 2021-08-04

**Authors:** Emeline Pierrieau, Jean-François Lepage, Pierre-Michel Bernier

**Affiliations:** 1 Département de Physiologie, Faculté de Médecine et des Sciences de la Santé, Université de Sherbrooke, Sherbrooke, Québec J1H 5N4, Canada; 2 Département de Pédiatrie, Faculté de Médecine et des Sciences de la Santé, Université de Sherbrooke, Sherbrooke, Québec J1H 5N4, Canada; 3 Département de Kinanthropologie, Faculté des Sciences de l’Activité Physique, Université de Sherbrooke, Sherbrooke, Québec J1K 2R1, Canada

**Keywords:** action selection, decision-making, effort, latency, reaching, reward

## Abstract

It is widely assumed that we select actions we value the most. While the influence of rewards on decision-making has been extensively studied, evidence regarding the influence of motor costs is scarce. Specifically, how and when motor costs are integrated in the decision process is unclear. Twenty-two right-handed human participants performed a reward-based target selection task by reaching with their right arm toward one of two visual targets. Targets were positioned in different directions according to biomechanical preference, such that one target was systematically associated with a lower motor cost than the other. Only one of the two targets was rewarded, either in a congruent or incongruent manner with respect to the associated motor cost. A timed-response paradigm was used to manipulate participants’ reaction times (RT). Results showed that when the rewarded target carried the highest motor cost, movements produced at short RT (<350 ms) were deviated toward the other (i.e., non-rewarded, low-cost (LC) target). In this context participants needed an additional 150-ms delay to reach the same percentage of rewarded trials as when the LC target was rewarded. Crucially, motor costs affected the total earnings of participants. These results demonstrate a robust interference of motor costs in a simple reward-based decision-making task. They point to the rapid and automatic integration of motor costs at an early stage of processing, potentially through the direct modulation of competing action representations in parieto-frontal regions. The progressive overcoming of this bias with increasing RT is likely achieved through top-down signaling pertaining to expected rewards.

## Significance Statement

Rapid evaluation of expected action costs for action selection possesses an adaptive value in ecological settings. The present work shows that these motor costs quickly and automatically bias decisions supposedly based on reward information, leading to lesser earnings when rewards and motor costs are incongruent. This bias is progressively overcome with increasing reaction times (RTs), consistent with the perspective of a hierarchical influence of different decisional variables on action representations based on their level of abstraction. Overall, these findings highlight the need to consider motor costs when using dynamic motor tasks for studying decision-making, especially under temporal pressure.

## Introduction

Should I run after the bus or wait for the next one? Should I grasp the pen on my right side or the one on my left? Motor decisions shape our daily life, allowing us to interact with our environment by selecting the actions we ultimately make (Cisek and Kalaska, 2010). Action selection is determined by optimization rules (Todorov and Jordan, 2002; Scott, 2012) to maximize a reward rate which defines the action value (Rangel and Hare, 2010; Carland et al., 2019). Hence action selection is often studied in paradigms manipulating action values, also called value-based decision-making. Value-based decision-making has mostly been investigated by varying the type, size, and probability of reward as well as the influence of time (Padoa-Schioppa and Assad, 2006; Kable and Glimcher, 2007; Klein-Flügge and Bestmann, 2012). Intriguingly, the involvement of the expected motor costs associated with each possibility of action, another fundamental parameter of the reward rate (Rangel and Hare, 2010; Carland et al., 2019), has been less studied and remains poorly understood, both at the behavioral (Morel et al., 2017) and neuronal levels (Walton and Bouret, 2019).

Understanding the integration of motor costs in the action selection process represents an important stake for the development of an ecological model of decision-making. Indeed, considering motor costs quickly and reliably is crucial when one has to flee from a predator or when hunting a prey. Inspired by these accounts, it has been suggested that sensorimotor representations of action possibilities, called affordances (Gibson, 1966), might be encoded and compete for action selection in parieto-frontal regions (Cisek, 2012; Gallivan et al., 2015; Pezzulo and Cisek, 2016). An intriguing possibility is that motor costs quickly modulate the early formation of action representations and/or the competition process taking place in parieto-frontal regions, and thus automatically bias action selection, even when it is supposed to rely on abstract or cognitive rules. This possibility is supported by recent behavioral studies demonstrating that motor costs strongly influence target selection in motor choices (Cos et al., 2011; Shadmehr et al., 2016; Gallivan et al., 2018) even when adding temporal pressure (Cos et al., 2014), and can also significantly bias perceptual-based judgements (Marcos et al., 2015; Hagura et al., 2017). Yet, these preceding studies have revealed a significant influence of motor costs only in uncertain perceptual decision-making contexts, that is when the information provided by visual stimuli is blurred and the perceptual decision harder. In these contexts, motor costs might have influenced decisions because of the lack of clear perceptual evidence favoring one of the options dictated by the abstract rule, thus making the less effortful option the most valued.

Hence, this evidence does not permit to disentangle if motor costs involve an automatic bias even when the decision is based on a clear and explicit abstract rule. To test that we developed a simple reward-based decision-making paradigm where the perceptual evidence remained the same across conditions and constant during trials. Critically, motor costs were manipulated by varying target positions in the workspace to influence the biomechanical complexity of the required reaching movement, and thus the amount of effort needed. We hypothesized that if motor costs automatically bias decisions, then there should be a significant influence of target position on choices although the level of perceptual evidence remains constant. We further assumed that this bias would be most apparent at short response latencies. In order to ensure a sufficient number of trials with short reaction times (RTs) and to better identify the latency at which motor costs might bias behavior, we used a timed-response task (Ghez et al., 1997; Cos et al., 2014; Haith et al., 2016). Results revealed that motor costs had a significant influence on participants’ behavior by impacting movement kinematics and target choices, and thus modulating the amount of rewards ultimately gained.

## Materials and Methods

### Participants

Twenty-two university students [10 females, 24 ± 4 (mean ± SD) years old] participated in this study. All participants had normal or corrected-to-normal vision. All were right-handed based on self-report and were free of any known neurologic or psychiatric condition. A $30 CAD compensation was given to participants ($15 per 1-h session) and they could earn up to an additional $20 CAD, depending on their performance. In all cases, participants finished the experiment with a net monetary gain averaging $44.5 ± 1.8. Participants gave their informed written consent, and all procedures were approved by the University of Sherbrooke institutional review board and ethics committee. The experiment conformed to the standards set by the 1964 Declaration of Helsinki.

### Experimental task

#### Set-up

The experimental setup consisted of a table supporting a 20-inch computer monitor that projected visual stimuli onto a mirror positioned horizontally in front of the participants. The monitor (Dell P1130 20-inch monitor; resolution: 1024 × 768; refresh rate: 150 Hz) was mounted face down 29 cm above the mirror and the mirror was positioned 29 cm above the table surface. A two-joint manipulandum composed of two lightweight metal rods with two potentiometers located at the manipulandum’s hinges permitted to record participants movements with an acquisition frequency of 100 Hz. Participants were asked to grasp a short handle located at the mobile end of the manipulandum, which position in the workspace was visible for the participants via a cursor projected on the monitor. Consequently, although participants could not see their right hand, they had constant visual feedback of the position of their hand, in a manner similar to a computer mouse. This set-up allowed participants to see the visual stimuli in the same plane as their hand and has already been used in published studies (Hamel et al., 2017; Hamel-Thibault et al., 2018; Savoie et al., 2019).

#### Overview

Participants were seated in front of this set-up. They were asked to reach toward visual targets (diameter: 3 cm) with their right hand. Their starting position was controlled by resting their chin on a small support and keeping their right elbow in contact with the surface of the table. They were also told to minimize postural changes during the experiment. To initiate a trial, participants had to place the cursor (white circle, diameter: 0.6 cm), and thus their hand, on a starting point located at the center of the screen (gray circle, diameter: 0.6 cm). Most of the trials (720/1200) were two-target (2T) trials that consisted of two targets located 90° apart on the screen, and the rest of the trials (480/1200) were one-target (1T) trials in which only one target was displayed. The difference between the two conditions is that in 2T participants had to choose which target they wanted to reach. There were four possible target locations: 60°, 150°, 240°, and 330°. All targets were at the same distance from the starting point (10 cm). In 2T, targets could appear upward (CONF1), leftward (CONF2), downward (CONF3), or rightward (CONF4) from the starting point. Each of these configurations contained one target located in a direction biomechanically easier to reach than the other (see below). The order of presentation of the trials was varied pseudo-randomly by ensuring that the same condition was not presented twice consecutively and that a 2T trial following a 1T trial did not consist of a configuration that included the same target as the one displayed in the 1T trial, to prevent repetitiveness of choices. Because the deliberation time was constrained (see below), the use of different configurations of targets provided stochasticity and prevented the stereotypical preplanning of any given movement. Six blocks of 200 test trials were used for each participant. The experiment was divided into two 1-h sessions (three blocks per session) separated by 24 h. During the first session, participants had to perform at least two blocks of 20 familiarization trials before beginning the experimental blocks. If they succeeded at correctly hitting targets in 15 out of 20 trials in the second block, they were allowed to move on with the first block of test trials, otherwise they had to perform another familiarization block of 20 trials.

#### Trial timeline

**Figure 1.**
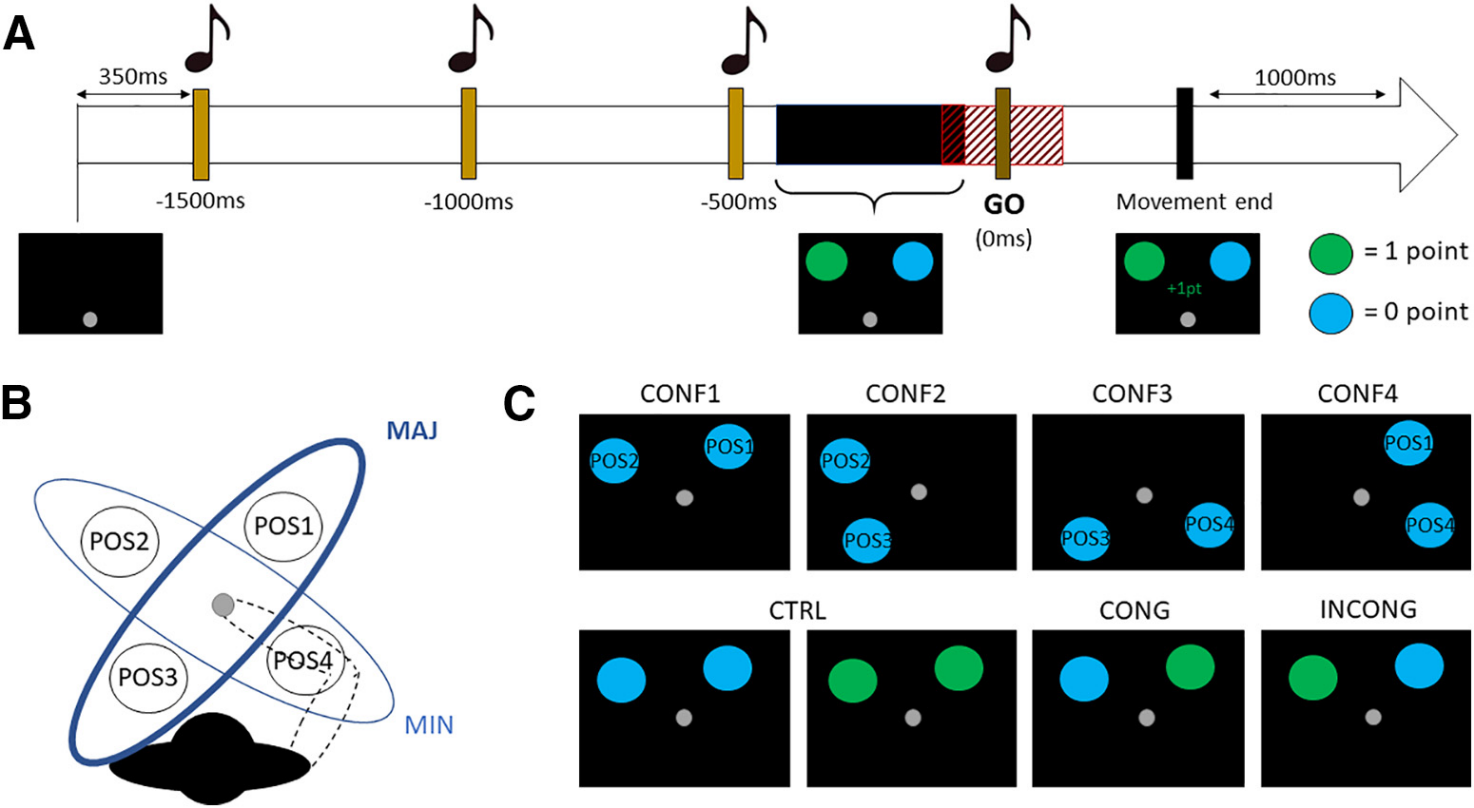
Experimental design. ***A***, Brown bars and musical notes indicate the four auditory cues. The black horizontal bar indicates the range of target onsets (−400 to –100 ms before go cue). The shaded red area around the go cue indicates valid movement onset interval (−150 to 150 ms). Reported times under the brown bars correspond to time differences from go cue (fourth tone). ***B***, LC targets on positions 1 (POS1 = 60°) and 3 (POS3 = 240°) were located on the major axis of the mobility ellipse (MAJ; thick blue ellipse) whereas HC targets on positions 2 (POS2 = 150°) and 4 (POS4 = 330°) were located on the minor axis of the mobility ellipse (MIN; thin blue ellipse). Dotted lines schematically illustrate the right arm initial position, and the gray circle indicates the hand initial position. ***C***, First row shows the four configurations of targets used in the task. Second row illustrates the control (CTRL; first and second panels), congruent (CONG; third panel), and incongruent (INCONG; fourth panel) conditions for the first configuration of targets (CONF1).

We used a timed-response task (Ghez et al., 1997; Cos et al., 2014; Haith et al., 2016) to control participants’ reach RTs. During each trial, participants heard a sequence of four rhythmic auditory tones separated by 500-ms intervals (Fig. 1*A*). The first tone was triggered after holding the cursor on the starting point for 350 ms. Targets were projected 100–400 ms before the fourth tone, according to a uniform distribution (60 trials per condition ranging from 100 to 400 ms with a 5-ms increment). Participants were told to initiate their movement as synchronously as possible with the fourth tone. Visual feedback was presented at movement end and remained for 1 s. In correct trials, the feedback indicated the number of points won (between 0 and 1 depending on MT when a green target was correctly reached or 0 when it was a cyan target, see below for further details). Trials in which participants initiated their movement >150 ms before or after the fourth tone were aborted and an error message was presented, informing them that they had lost one point because they were too fast or too slow. The diameter of the targets was relatively large (3 cm) to minimize a potential precision bias which could interfere with the influence of biomechanical costs on decisions (Cos et al., 2012). Accordingly, there was no penalty for missed-target trials, an error message simply indicated that the participant had won no point because the target was missed. Once the movement ended, participants had to bring the cursor back to the starting point to initiate the next trial.

#### Manipulation of rewards and costs

Each reaching movement has a biomechanical cost that depended on the direction of the movement in regard to the ellipse of mobility of the right arm in our task. Indeed, previous studies showed that targets located on the major axis of the ellipse of mobility (60° and 240°) are chosen more often than targets located on the minor axis (150° and 330°), because their associated reaching movement carries a lower biomechanical cost (Cos et al., 2011; Shadmehr et al., 2016; Michalski et al., 2020; Fig. 1*B*). Importantly, in each 2T configuration one target was located on the major axis of the ellipse of mobility of the participant’s arm and thus associated with a low biomechanical cost (LC), whereas the other was located on the minor axis and considered as a high-cost (HC) target. Hence, there was a difference of motor costs between the two action possibilities. Participants were not told that some targets were more costly to reach than others. Targets could appear green or cyan, with an equal level of luminance. Participants were told that green targets were rewarded (one point) whereas cyan targets were not, and that the cumulated points would be converted into net earnings at the end of the experiment (42 points = $1 CAD). Additionally, to incentivize speed, the magnitude of reward on a trial gradually decreased as a function of movement time (MT); if the MT was above 250 ms, the reward was decreased by 0.2 points per additional 100 ms. Thus, in 2T condition, the manipulation of reward and motor cost could be congruent (CONG) when the LC target was green (rewarded) and the HC target was cyan (non-rewarded), or on the contrary it could be incongruent (INCONG) when the HC target was green and the LC target was cyan. There also was a control condition (CTRL) in which both targets were the same color, so that the participants’ decision should be based exclusively on the difference in motor costs between targets (Fig. 1*C*).

### Data analysis

Visual stimuli were presented using Psychtoolbox on MATLAB (MathWorks). Hand position was estimated in real time with the coordinates of the two potentiometers in the workspace. Movement onset was defined as the first time point when the coordinates of the hand were outside the starting point. Movement end was defined as the first time point when the coordinates of the hand were recorded inside of one of the presented targets with a velocity below one pixel per second. Trials where movement velocity fell below one pixel per second outside of the presented targets were considered as missed-target trials. RTs were calculated as the latency between target appearance and movement onset. MTs were calculated as the latency between movement onset and movement end. In 2T trials, the target where the movement ended was considered as the final choice of participants. Importantly, because of the possibility of rapid influence of motor costs on choices (Cos et al., 2014) and changes-of-mind during movement (Resulaj et al., 2009), we investigated the initial choice of participants. The initial choice was determined according to which quadrant the hand was located 100 ms after movement onset. Missed-target trials where the hand position at movement end was <1 cm around the edges of one of the targets (<4 cm from the target center) were kept for further analysis. Indeed, because there was a consequent proportion of missed-target trials (∼9%) because of the high time pressure, and because we were mainly interested in target choices and not in movement accuracy, we considered that movements ending <1 cm around a target indicated that this target was chosen by the participant. Participants had to initiate their movements in a time window of 300 ms centered on the go cue (fourth tone) or else the trial was aborted and they lost one point (see above). Hence, these error trials were also excluded from the analysis. 2T trials for which the trajectory angle 100 ms after movement onset was outside of the quadrants containing the targets (±45° from the target) were excluded from analysis (0.3% of 2T trials) to ensure that the observed action was not the result of a default or preplanned response. 1T trials for which the trajectory angle 100 ms after movement onset was >90° from the target were excluded (0.2% of 1T trials). The difference in accuracy criterion between 1T and 2T trials was chosen to make both conditions comparable. Indeed, we reasoned that to control whether a rapid bias toward the LC direction was the result of a preplanned movement or a deviated trajectory to reach the HC target, we should keep 1T trials in which the trajectory would have been deviated in these directions.

### Experimental design and statistical analysis

All analyses were conducted on the 22 participants that took part in the experiment. In order to take into account interindividual variability in behavior, we used general linear mixed models (GLMMs) instead of ANOVA for within-subjects comparisons. Indeed, GLMM allow to analyze data with different numbers of observations per subject and condition by assigning participants as a random factor in the model, making it a robust approach for the analysis of biological data (Harrison et al., 2018). For each analysis we ran several models including the different combinations of fixed and random factors and interactions between them. We then selected the model with the lowest Akaike Information Criterion (AIC). AIC is a statistic that quantifies the loss of information resulting from modeling the real process underlying the data by taking into account concurrently the bias and the variance of the model (Symonds and Moussalli, 2011). Importantly, all GLMM that we used included subject (22 levels: one for each participant) as a random variable. We used paired *t* tests or Wilcoxon tests if the data were not normally distributed (*p* < 0.05 Shapiro–Wilk test) for pairwise comparisons. A Bonferroni correction of *p* values was applied when conducting multiple pairwise comparisons. For each statistical test conducted, Cohen’s *d* was reported to indicate effect size (Lakens, 2013). Statistical analyses were computed using Jamovi v.1.2.27 (the jamovi project, 2019, *Jamovi*, computer software, retrieved from https://www.jamovi.org), a software that implements R statistical language (R Core Team, 2018, R: a language and environment for statistical computing, computer software, retrieved from https://www.cran.r-project.org/).

## Results

**Figure 2.**
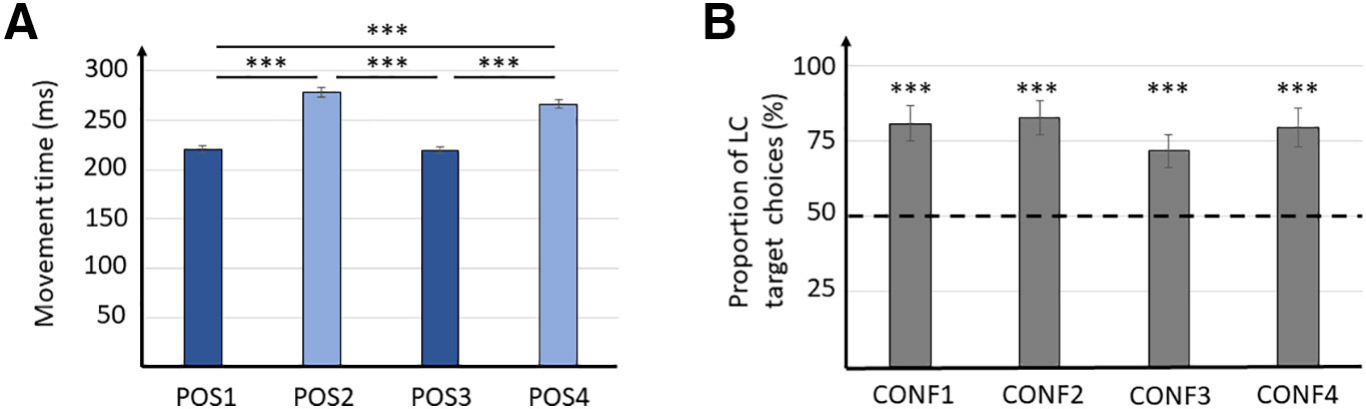
Effects of motor costs on movements and choices without rewards. ***A***, Average MT for each target position in 1T trials. ***B***, Average proportion of LC target choices (targets in positions 1 and 3), for each target configuration. Values higher than 50 demonstrate a bias toward the LC target. Error bars indicate ci95 around the mean; *p < 0.05, **p < 0.01, ***p < 0.001.

We first sought to verify whether motor costs had a significant influence on participants’ movements and choices when targets were equally valued. To do so, we isolated both 1T and 2T trials in the CTRL condition. Targets in positions 1 and 3 were located on the major axis of the ellipse of mobility and thus associated with a LC, whereas targets in positions 2 and 4 were on the minor axis and considered as HC targets (see Materials and Methods; Fig. 1*B*). Targets were rewarded or not depending on their color (cyan: no reward, green: reward). GLMM that included position (four levels: POS1, POS2, POS3, and POS4) and color (two levels: cyan or green) were conducted on MT in 1T trials. The selected GLMM [MT ∼ 1 + position + color + position:color + (1 + position | subject), AIC = −36 593.1, BIC = −36 373.8, marginal *R*^2^ = 0.23, conditional *R*^2^ = 0.53] showed a significant effect of the position of the target on MT (*F*_(3,21)_ = 128.0, *p* < 10^−5^), but neither an effect of color (*F*_(1,9847.7)_ = 0.03, *p* = 0.858) nor an interaction between them (*F*_(3,9847.9)_ = 0.8, *p* = 0.519). *Post hoc* analyses showed that MT in 1T trials were significantly lower for movements directed to target position 1 compared with targets located in position 2 (*t*_(21)_ = −15.4, *p* < 10^−5^, Cohen’s *d* = −3.3) and position 4 (*t*_(21)_ = −14.3, *p* < 10^−5^, Cohen’s *d* = −3.1). Similarly, movements directed to target in position 3 were significantly faster than those directed to targets in position 2 (*t*_(21)_ = −16.2, *p* < 10^−5^, Cohen’s *d* = −3.5) and position 4 (*t*_(21)_ = −12.8, *p* < 10^−5^, Cohen’s *d* = −2.7), thus globally showing that MT was reduced when reaching to LC as compared with HC targets (Fig. 2*A*).

In 2T trials, the potential decisional bias incurred by motor costs was assessed using one-sample Wilcoxon-ranked tests on the average proportion of LC target choices across participants in the CTRL condition for each configuration of targets. Precisely, the statistical test aimed to determine whether the proportion of LC target choices was significantly different from 50%. Results showed that participants’ choices were significantly biased toward the LC targets, regardless of the configuration (CONF1: *W*_(21)_ = 253, *p* < 10^−5^, Cohen’s *d* = 2.3; CONF2: *W*_(21)_ = 243, *p* = 10^−4^, Cohen’s *d* = 1.7; CONF3: *W*_(21)_ = 238, *p* = 10^−4^, Cohen’s *d* = 1.2; CONF4: *W*_(21)_ = 253, *p* < 10^−5^, Cohen’s *d* = 2.0; Fig. 2*B*). Note that participants tended to choose more often the LC target in 2T rewarded trials compared with non-rewarded trials (*t*_(21)_ = 3.3, *p* = 0.003, Cohen’s *d* = 0.7), but the mean difference between choices was low (3.8%) and was not sufficient to significantly impact the average MT between rewarded and non-rewarded trials (*t*_(21)_ = 0.4, *p* = 0.672). Furthermore, a control analysis conducted only on non-rewarded CTRL trials also showed a significant preference for LC targets (CONF1: *W*_(21)_ = 238, *p* = 10^−4^, Cohen’s *d* = 1.3; CONF2: *W*_(21)_ = 249, *p* = 10^−4^, Cohen’s *d* = 1.9; CONF3: *W*_(21)_ = 247, *p* = 10^−4^, Cohen’s *d* = 1.7; CONF4: *W*_(21)_ = 249, *p* = 10^−4^, Cohen’s *d* = 1.9). These first results confirm that participants’ movements and choices were significantly influenced by the differential motor costs associated with each target in our task, which replicates previous findings using similar tasks (Cos et al., 2011, 2014; Shadmehr et al., 2016).

**Figure 3.**
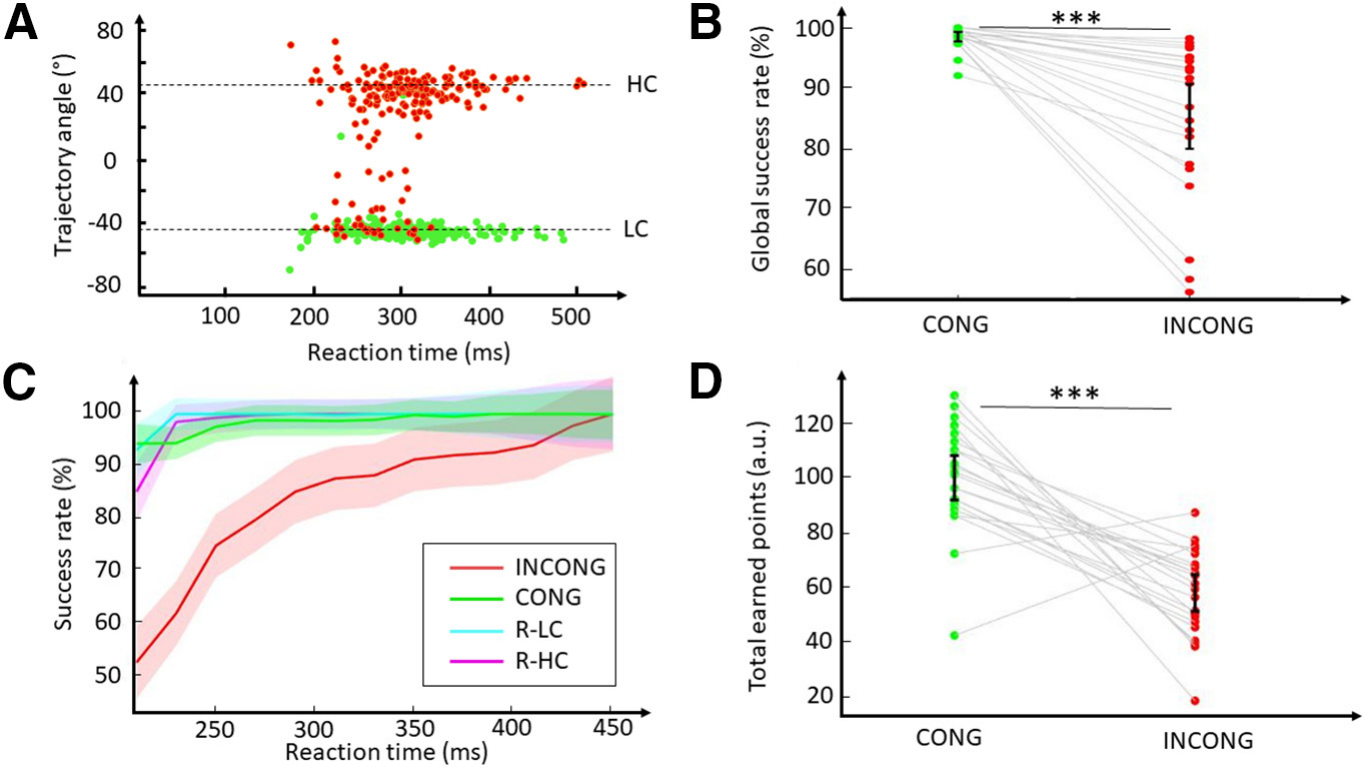
Effects of motor costs on reward-based decision-making. ***A***, Distribution of trajectory angles 100 ms after movement onset as a function of RT in CONG (green dots) and INCONG (red dots) conditions for a representative participant in CONF1. Dotted lines indicate the angle of the LC target and HC targets. ***B***, Global success rates based on initial choices (proportion of trials for which the hand location 100 ms after movement onset was in the quadrant of the rewarded target) in CONG and INCONG conditions for each participant. ***C***, Success rates as a function of RT in CONG (green line), INCONG (red line), R- LC (cyan line), and R-HC (magenta line) conditions. The shaded areas indicate ci95 around the mean. ***D***, Total earned points during the experiment in CONG and INCONG conditions for each participant. Error bars indicate ci95 around the mean; *p < 0.05, **p < 0.01, ***p < 0.001.

The next step of the analyses consisted in testing the influence of motor costs on reward-based decisions. Specifically, we aimed to compare the accuracy of the decisions in CONG and INCONG conditions. We analyzed initial choices by calculating the trajectory angle 100 ms after movement onset and comparing it with the actual angle of the rewarded target in each condition (Fig. 3*A*). The success rate was defined as the proportion of trials oriented toward the quadrant of the rewarded target 100 ms after movement onset. Consequently, trials with movements initiated (100 ms after movement onset) in the quadrant of the rewarded target were considered as correct and trials with movements initiated in the quadrant of the non-rewarded target were considered as incorrect. Globally, participants were less accurate in INCONG than in CONG trials, their success rates being lower in the former condition (*W*_(21)_ = 253, *p* = 10^−4^, Cohen’s *d* = 1.1; Fig. 3*B*). In order to probe the magnitude of this difference for the different deliberation periods, we then computed the success rates of participants according to their RT. As expected, the success rates increased with the length of RT, but this rise appeared slower in the INCONG condition than in the CONG condition (Fig. 3*C*). Because we used the hand trajectory to define choices, we controlled for any default bias that could be because of the position of the target by adding 1T trials to the model. More specifically, we used rewarded 1T trials, split according to their motor cost (R-HC and R-LC). Thus, the only difference between INCONG and R-HC trials was the presence of the non-rewarded LC target in the INCONG condition (in the same way the presence of the non-rewarded HC target in the CONG condition when comparing CONG and R-LC trials). The analysis demonstrated that success rates were significantly modulated both by conditions and RT (Fig. 3*C*). We used a GLMM with condition (four levels: CONG, INCONG, R-LC, and R-HC) and RT (13 levels: 20-ms bins ranging from 200 to 460 ms) as fixed factors. The lower bound of RT analysis was fixed at 200 ms to ensure the validity and representativity of the observed behavior, because of the lack of datapoints in 20-ms bins below this time (only 15 trials comprised between 180 and 200 ms originating from seven of the 22 participants). This might be explained by the shortest stimulus-response interval fixed (100 ± 150 ms), allowing maximal RT of 250 ms. In this context, it was optimal to wait as much as permitted to fully process the position and color associated with the targets to reach the rewarded one, instead of initiating the movement too early and missing the reward. The analysis [success rates ∼ 1 + condition + RT + condition:subject + (1 +  condition | subject), AIC = −3516.7, BIC = −2827.8, marginal *R*^2^ = 0.31, conditional *R*^2^ = 0.46] showed significant effects of condition (*F*_(3,56.9)_ = 10.2, *p* = 10^−5^) and RT (*F*_(12,2481.0)_ = 22.9, *p* < 10^−5^) on success rates and an interaction between condition and RT (*F*_(36,2481.1)_ = 10.5, *p* < 10^−5^). Crucially, the difference in success rates between CONG and INCONG trials was significant until the RT reached [340, 360 ms] ([200, 220 ms]: *t*_(108.8)_ = 10.6, mean diff = 41.5%, *p* < 10^−5^; [220, 240 ms]: *t*_(57.9)_ = 9.7, mean diff = 32.2%, *p* < 10^−5^; [240, 260 ms]: *t*_(51.9)_ = 6.9, mean diff = 22.5%, *p* < 10^−5^; [260, 280 ms]: *t*_(51.9)_ = 5.8, mean diff = 18.8%, *p* = 10^−4^; [280, 300 ms]: *t*_(51.9)_ = 4.2, mean diff = 13.5%, *p* = 0.002; [300, 320 ms]: *t*_(51.9)_ = 3.2, mean diff = 10.9%, *p* = 0.018; [320, 340 ms]: *t*_(51.9)_ = 3.2, mean diff = 10.5%, *p* = 0.027; [340, 460 ms]: *t* < 2.6, mean diff < 8.4%, *p* > 0.160).

Another way of assessing the difference in the accuracy of choices between CONG and INCONG conditions is to use an absolute value of success rate (95%) as a criterion; 95% confidence intervals (ci95) around the mean of success rates for each range of RT included the value 95 as early as the lowest range of RT in the CONG condition ([200, 220 ms], mean = 94.4%, ci95 = [90.7, 98.2]), whereas in the INCONG condition participants did not reach this success rate until RTs of 350 ms ([340, 360 ms], mean = 91.4%, ci95 = [85.4, 97.4]). This observation is consistent with the previous results using GLMM. Overall, these results show that participants needed an additional delay of ∼150 (140–160) ms to achieve a similar success rate when the HC target was rewarded compared with when the LC target was rewarded, suggesting considerable interference of motor costs on reward-based choices.

**Figure 4.**
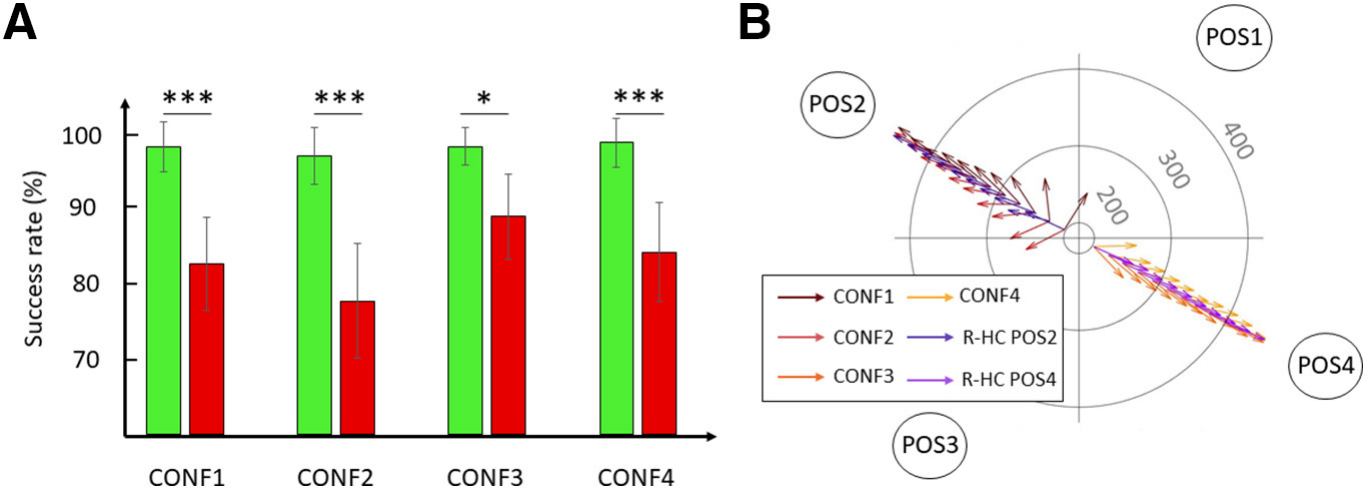
Influence of target configuration on directional bias. ***A***, Average success rates according to configurations of targets and congruence (green bars, CONG; red bars, INCONG). Error bars indicate ci95 around the mean; *p < 0.05, **p < 0.01, ***p < .001. ***B***, Mean trajectory angle 100 ms after movement onset in INCONG condition for CONF1 (brown arrows), CONF2 (red arrows), CONF3 (orange arrows), and CONF4 (yellow arrows), and in R-HC condition for POS2 (dark purple arrows) and POS4 (light purple arrows). Concentric circles indicate RT (gray values in ms).

Critically, this difference in success rates between CONG and INCONG conditions was not explained by a difference between trajectories needed to reach the LC and the HC target. Indeed, there was no significant difference between R-HC and R-LC in success rates at any RT tested ([200, 460 ms]: *t* < 2.1, mean diff < 7.9 ms, *p* > 0.530). Additionally, the choice bias observed in the INCONG condition did not appear to result from a default movement, made without considering the presented targets at short RT, because we noted a significant difference in success rates between INCONG and R-HC over RT ranging from 200 to 340 ms, comparable to the difference previously found between CONG and INCONG conditions ([200, 220 ms]: *t*_(169.1)_ = 7.2, mean diff = 32.3 ms, *p* < 10^−5^; [220, 240 ms]: *t*_(61.2)_ = 10.5, mean diff = 36.3 ms, *p* < 10^−5^; [240, 260 ms]: *t*_(50.7)_ = 7.4, mean diff = 24.2 ms, *p* < 10^−5^; [260, 280 ms]: *t*_(50.1)_ = 6.1, mean diff = 19.9 ms, *p* = 10^−5^; [280, 300 ms]: *t*_(50.1)_ = 4.5, mean diff = 14.7 ms, *p* = 10^−4^; [300, 320 ms]: *t*_(50.1)_ = 3.7, mean diff = 12.2 ms, *p* = 0.007; [320, 340 ms]: *t*_(50.1)_ = 3.6, mean diff = 11.6 ms, *p* = 0.011) until [340, 360 ms] ([340, 460 ms]: *t* < 2.7, mean diff < 8.7 ms, *p* > 0.156). This suggests that the shift in the initial trajectory observed in the INCONG condition was specifically due to the presence of the non-rewarded LC target (Fig. 3*C*). The difference in initial choices between INCONG and R-HC conditions was also observable in the average angle of the initial trajectory at shortest RT (Fig. 4*B*).

The next question we asked was whether this bias in initial choice was further corrected or not. We compared initial and final choices of participants in INCONG trials across RT ranges. The GLMM included two fixed factors: choice (two levels: initial and final) and RT (13 levels: 20-ms bins ranging from 200 to 460 ms). The selected model [success rates ∼ 1 + choice + RT + choice:RT + (1 +  choice + RT | subject), AIC = −621.5, BIC = 206.5, marginal *R*^2^ = 0.19, conditional *R*^2^ = 0.60] demonstrated a significant effect of RT (*F*_(12,30.1)_ = 9.2, *p* < 10^−5^) and a significant effect of choice (*F*_(1,96.4)_ = 6.7, *p* = 0.01) but no interaction between RT and choice (*F*_(12,1226.2)_ = 1.0, *p* = 0.441). The magnitude of the choice effect was relatively low because success rates were only on average 2.6% [ci95: 0.7%, 4.5%] higher in final choices compared with initial choices. Additionally, the absence of interaction between choice and RT suggests that initial and final choices evolved in a similar manner as a function to RT. Consequently, at shorter latencies participants’ initial choices were not only biased in the quadrant of the LC target, but their final choice also corresponded more often to the non-rewarded target. This tendency was confirmed by the analysis conducted on the total earned points. We first removed the MT-based correction (no decrease in the number of earned points according to MT) so that the total amount of points was not influenced by the longer MT needed to reach to the rewarded target in INCONG than in CONG conditions (Fig. 2*A*). The analysis showed that participants won significantly more points in the CONG than in the INCONG trials (*t*_(21)_ = 6.4, mean diff = 42.3, *p* < 10^−5^, Cohen’s *d* = 1.4; Fig. 3*D*). Hence, the difference in motor costs between targets meaningfully impacted the total earnings of participants.

Beside motor costs, other factors might have also biased choices in the present task. Namely, low-level visuo-attentional processes may also have had an influence. These preferences possibly involve a right-hemifield visual bias for right-handed individuals as well as for movements performed with the right hand (Coelho et al., 2013; Le and Niemeier, 2014). In order to test for such visuo-attentional bias, we analyzed the difference in success rates between CONG and INCONG conditions separately for each configuration of targets. We included only trials with RT < 350 ms because it was at these latencies that motor costs significantly influenced participants’ choices in previous analyses (see above). The GLMM included congruence (CONG, INCONG) and configuration (CONF1, CONF2, CONF3, CONF4) as fixed factors [success rates ∼ 1 + congruence + configuration + congruence:configuration + (1 + congruence + configuration | subject), AIC = −766.3, marginal *R*^2^ = 0.23, conditional *R*^2^ = 0.62]. It demonstrated a significant effect of congruence (*F*_(1,21.1)_ = 28.6, *p* = 10^−5^), configuration (*F*_(3,26.3)_ = 5.1, *p* = 0.007) as well as an interaction (*F*_(3,457.0)_ = 5.6, *p* = 0.001). *Post hoc* analyses revealed significant differences in success rates between CONG and INCONG for CONF1 (*t*_(35.7)_ = 5.0, mean diff = 15.8%, *p* = 10^−5^), CONF2 (*t*_(35.7)_ = 6.1, mean diff = 19.3%, *p* < 10^−5^), and CONF4 (*t*_(35.7)_ = 4.6, mean diff = 14.6%, *p* = 10^−4^). This effect was also significant for CONF3 but was of smaller magnitude (*t*_(35.7)_ = 2.9, mean diff = 9.3%, *p* = 0.02; Fig. 4*A*). This smaller effect for CONF3 suggests that a rightward visual bias could have countered the influence of motor costs, because in this condition the LC target was located on the left hemifield whereas the HC target was located on the right hemifield. Comparisons between configurations of targets showed no significant difference in success rates in CONG condition (*t* < 0.5, mean diff < 1.3, *p* = 1), whereas success rates in INCONG were higher in CONF3 compared with CONF1 (*t*_(46.8)_ = 3.0, mean diff = 6.5%, *p* = 0.024) and CONF2 (*t*_(43.8)_ = 5.1, mean diff = 11.3%, *p* = 10^−5^). Hence, choices in INCONG condition were not equivalent between configurations of targets, especially comparing CONF2 and CONF3. Importantly, only the position of the HC target differed between both conditions (Fig. 1*C*), suggesting that this behavioral effect should be attributable to a different degree of preference for HC targets depending on their locations. Indeed, the choice was significantly more biased toward the LC target when the HC target was located in the upper left quadrant (CONF1 and CONF2) compared with when it was located in the lower right quadrant (CONF3 and CONF4; Fig. 4*B*). Overall, these results suggest that a rightward visual bias could have influenced initial choices along with motor costs. Nonetheless, the lack of significant preference for the LC target in CONF1 as compared with other target configurations limits this interpretation. Hence, the existence of an early visual bias remains to be clarified. Finally, the last part of the analysis aimed to determine whether motor costs were learned and thus had a growing influence on participants’ choices over the course of the experiment, or whether they consisted in a bias that was already present at the beginning of the experiment. The GLMM included two fixed factors: congruence (two levels: CONG and INCONG) and block (six levels). The analysis [success rates ∼ 1 + congruence + block + (1 + congruence | subject), AIC = –1067.7, BIC = −915.2, marginal *R*^2^ = 0.16, conditional *R*^2^ = 0.44] revealed a significant effect of congruence on participants’ choices (*F*_(1,20.9)_ = 23.9, *p* = 10^−5^) but neither a significant effect of block (*F*_(5,866.1)_ = 0.8, *p* = 0.544) nor an interaction between congruence and block (*F*_(5,866.1)_ = 1.2, *p* = 0.298). The presence of a bias in choices between CONG and INCONG as early as the first block and the absence of a significant change in this bias across blocks suggest that the influence of motor costs on behavior was not acquired during the experiment.

## Discussion

This study revealed that varying the relative positions of targets, and thus the motor costs associated with each movement, is enough to influence a decision based on simple visual cues specifying rewards. Precisely, motor costs significantly biased initial choices, represented by the direction of the hand trajectory 100 ms after movement onset, when RT ranged from 200 to 350 ms. This bias seemed to delay the normativity of the decision because it took ∼150 ms more to achieve a similar success rate when the rewarded target was the most biomechanically costly (INCONG) compared with when the rewarded target carried the lowest cost (CONG). Motor costs had a substantial impact since participants earned significantly less reward in the INCONG than in the CONG condition. Importantly, the bias in INCONG initial trajectory could not be explained as an intrinsic feature of the trajectory used to reach the HC targets, since this deviation was not observed in 1T trials involving a HC rewarded target (R-HC).

These results should be interpreted keeping in mind that reward information was varied in the simplest way in our task with a binary color-based choice (for details, see Materials and Methods). The stimuli were equiluminant, there was no perceptual ambiguity between them, and the perceptual evidence remained constant throughout the trial. Additionally, participants were not explicitly informed that some targets would be easier to reach than others; they were only told that green targets were associated with points and that whatever monetary gain they accumulated by the end of the experiment would be theirs. A “normative” decision in this task should thus only consider expected rewards and not motor costs since the latter were irrelevant to the task. In this light, the fact that motor costs impacted time-constrained choices (impeding success rates), speaks to the automatic nature of their influence. This extends previous studies reporting a significant influence of motor costs on effort-based (Cos et al., 2014; Gallivan et al., 2017; Morel et al., 2017) and perceptual-based decision-making (Marcos et al., 2015; Hagura et al., 2017). This influence is particularly relevant to underline because the trajectory of reaching movements has been frequently used to infer choices based on visual and cognitive information (for review, see Song and Nakayama, 2009; Gallivan et al., 2018) even in high-speed decision contexts (Chapman et al., 2010, 2015; Carroll et al., 2019), but little interest has been given to the impact of motor costs carried by the different targets in these contexts.

There is a debate between serial and parallel models regarding the functional architecture underlying decision-making (Wispinski et al., 2020). In short, serial models state that decisions are made in a space of goods representing abstract values of options in the prefrontal cortex (Padoa-Schioppa, 2011), whereas parallel models suggest that decisions are made in a space of actions through a competition between sensorimotor representations of actions in parieto-frontal regions (Cisek and Kalaska, 2010). This debate is a central issue in the understanding of the integration of motor costs in the decision process because there is evidence that motor costs might be integrated with reward information in the prefrontal cortex (Cai and Padoa-Schioppa, 2019), but also that they might bias the decision quickly and thus be rather integrated in sensorimotor regions (Cos et al., 2014; Christopoulos and Schrater, 2015; Gallivan et al., 2017). The present results do not allow to resolve this debate, because they can be explained by both models. Indeed, in our task the success rate was not significantly different from 50% in the INCONG condition at the shortest RTs (Fig. 3*C*), meaning that participants might have considered both reward and motor cost information before initiating their movements. Consequently, motor costs could have increased the conflict in a competition between target values, or conversely, they could have biased action representations while being modulated by top-down signals specifying reward information. Nonetheless, the present study highlights the importance of considering basic motor costs inherent to reaching in different directions, even in a context in which target choices supposedly rely on other variables.

Previous studies have shown that motor costs influence decisions in <200 ms (Cos et al., 2014), with activation of sensorimotor regions related to the evaluation of motor costs found as early as 100 ms after stimulus onset (Harris and Lim, 2016). These data, in line with ours, suggest that there appears to be no RT that is too fast for motor costs not to be considered. This would be consistent with the rapid formation of sensorimotor representations of action possibilities (Cisek and Kalaska, 2010), and point to the possibility that motor costs might bias action representations at a very early stage. More specifically, reaching movements are thought to be represented within directionally-tuned neuronal assemblies in the dorsal parieto-frontal cortex, as a result of the integration of arm-related and target-related sensory signals (Buneo et al., 2002; Pesaran et al., 2006; Bernier et al., 2017). An intriguing possibility is that motor costs are intrinsically factored in this arm-target integration process. This could take the form of a gain on the activity of directionally-selective neurons when the reach direction incurs low motor costs. Such “native” biasing of action representations according to cost may be akin to a subset of these regions responding preferentially to targets in peripersonal space (i.e., at a reachable distance; Gallivan et al., 2009, 2011). This early bias has been suggested for attentional and other cognitive biases in perception, under the concept of priority maps (Andersen and Cui, 2009; Roggeman et al., 2010; Klink et al., 2014). A unifying mechanism underlying visuo-attentional preferences and the influence of motor costs would also explain why they both influenced choices at the shortest RTs in our task. Indeed, participants were more accurate when the rewarded HC target was located in the right visual hemifield as compared with when it was located in the left hemifield (see Fig. 4*B*). This observation is consistent with previous studies that demonstrated an ipsilateral hemifield preference for movements performed with a given hand (Coelho et al., 2013; Le and Niemeier, 2014). Still, the motor cost bias remained significant in all tested configurations of targets, suggesting that it was robust in our task.

Alternatively, motor costs might be computed in other cortical and subcortical regions that influence parieto-frontal activity. It may arise from inputs from the basal ganglia and the cerebellum, which are known to modulate activity in sensorimotor regions by means of cortico- subcortical loops (Pezzulo and Cisek, 2016). Previous studies suggested that motor costs could influence action selection by the re-activation of a stored internal model of limbs biomechanics in the cerebellum (Dounskaia, 2005; Goble et al., 2007). This is consistent with the proposed role of the cerebellum in motor learning and prediction of sensory consequences of movement (Shadmehr and Krakauer, 2008). Additionally, recent studies highlighted the involvement of cortico-striatal circuits in the evaluation of effort mediated by dopamine, including the medial frontal cortex and the dorsal striatum (Kurniawan et al., 2010; Prévost et al., 2010; Zénon et al., 2015; Klein-Flügge et al., 2016). However, the role of dopamine in effort encoding is currently a debated topic (Salamone et al., 2016; Walton and Bouret, 2019), and the extent to which our results are linked to these preceding studies remains to be determined. Indeed, most of them have used hand grip tasks in which participants had to assess the cost associated with each level of isometric contraction and compare it with an expected reward by means of explicit, conscious computations (Prévost et al., 2010; Zénon et al., 2015; Klein-Flügge et al., 2016; Chen et al., 2020). This is in contrast with our task where motor costs inherent to reaching movements are arguably assessed more implicitly, notably because of the absence of a stimulus indicating the level of required effort. Moreover, varying the force or duration of an isometric contraction results in only manipulating the energetic cost of the movement, whereas the biomechanical preference for reaching in specific directions appears mainly driven by a simplification of neural control (Goble et al., 2007). It constitutes a notable consideration because motor costs are defined as a combination of an energetic cost and a control cost (Shadmehr and Krakauer, 2008), and the perception of effort appears not to rely only on the computation of an energetic cost (Morel et al., 2017).

One limitation concerning the interpretation of the results is a potential preexisting influence of motor costs on action selection, before processing target information. This concern is related to previous studies that have shown that movements are biased toward the lowest cost directions in a context where there is no target to reach (Wang and Dounskaia, 2012) or movements have to be initiated before target onset (Haith et al., 2016). Consequently, an alternative explanation of the rapid motor cost influence observed in the present results is that this influence preexisted the processing of target, and thus was independent of the position of the presented targets. However, this preexisting bias appears unlikely in our task for several reasons. First, if this initial bias was preexisting, we should have seen it also in the 1T trials directed toward the HC target at the same RT. However, as detailed in Results, there was no such bias. Second, if the bias was preexisting, there should be movements initiated in a quadrant where no target was displayed. However, there were very few of those (0.3% of the trials, see Materials and Methods). Furthermore, it should be noted that we used four different configurations of targets and alternated randomly 2T and 1T to ensure stochasticity. In this context, participants could not anticipate where the targets would appear, restricting their capacity to preplan their movements. Finally, participants appeared to wait as much as permitted before initiating their movements (few trials with RT < 200 ms, see Results), indirectly suggesting that they tried to process target information.

In conclusion, our results suggest that motor costs bias action selection even in a reward- based decision-making context, possibly by providing an early boost to action representations associated with lower motor costs. Consequently, when reward information is incongruent with motor costs, this initial bias would have to be overcome by the gradual accumulation of evidence in favor of the other rewarded action, thus accounting for the observed 150-ms delay. This increasing (albeit slower) consideration of the associated reward is likely to be because of top- down signaling from prefrontal cortex and basal ganglia, regions known to be involved in the computation of stimulus-reward association rules (Sleezer et al., 2016; Ebitz et al., 2020). Overall, these findings are in line with the perspective of a hierarchical influence of different decisional variables on action representations, based on their level of abstraction (Cisek, 2012; Pezzulo and Cisek, 2016). This underlines the importance of taking motor costs into consideration when using dynamic motor tasks for studying decision-making and to further investigate the underlying neural basis of the integration of motor costs in the action selection process.

## Synthesis

Reviewing Editor: Mark Laubach, American University Decisions are customarily a result of the Reviewing Editor and the peer reviewers coming together and discussing their recommendations until a consensus is reached. When revisions are invited, a fact-based synthesis statement explaining their decision and outlining what is needed to prepare a revision will be listed below. The following reviewer(s) agreed to reveal their identity: Giuseppe Pellizzer. Note: If this manuscript was transferred from JNeurosci and a decision was made to accept the manuscript without peer review, a brief statement to this effect will instead be what is listed below.

Both reviewers agreed that the revisions improved the manuscript and had no further concerns.
